# Identifying factors that influence electric vehicle charging station performance in expanding networks

**DOI:** 10.1371/journal.pone.0302132

**Published:** 2024-04-26

**Authors:** Rick Wolbertus

**Affiliations:** Department of Urban Technology, Faculty of Technology, Amsterdam University of Applied Sciences, Amsterdam, The Netherlands; UCL: University College London, UNITED KINGDOM

## Abstract

Charging infrastructure deployment has taken off in many cities with the rise of the number of electric vehicles on the road. Expansion of infrastructure is a matter of prioritisation of resources to optimise the infrastructure. This paper explores how to measure charging station performance, to address the challenges that policy makers face. These performance indicators are used in a regression model, based upon current utilisation of the network, to predict which charging stations perform best. The results show that a model based on available geographical data and performance metrics of the current network are best combined to predict infrastructure performance. The variability between public charging stations is however big, as frequent user characteristics do determine the performance to a large extent.

## 1. Introduction

Electric vehicles (EVs) have reached significant market shares in a multitude of countries in recent years. The market develops from early adopters to mainstream consumers as the range of vehicles increases and prices decrease [1]. Increasingly stringent regulations in markets such as the European Union push most Original Equipment Manufacturers (OEMs) to announce more electric models to be launched in the near future [2]. OEMs make plans to completely phase out their gasoline portfolio within a decade [3]. Alongside the rise of electric vehicles, charging infrastructure has been developed in many countries to accommodate for the growth in the number of vehicles. Despite initial infrastructure development, there are still significant worries if the roll out can keep up pace with the adoption pace of vehicles.

Sufficient charging infrastructure is considered the major bottleneck for a swift transition to electric mobility [4, 5]. EV charging is known to mostly take place at home, workplace and public locations [6, 7]. For those without a private charging opportunity, public on-street charging is a substitute for home and workplace charging. Especially dense urban areas need sufficient public charging infrastructure as the majority of parking is done on-street [8]. Realisation of charging infrastructure in cities requires to balance different aspects such as public space, parking, business cases and electricity grid integration [9, 10]. Policy makers face the problem that the definition of charging infrastructure success is not one-dimensional, which in turn makes it difficult to capture and assess its performance.

Research on the topic of charging infrastructure networks has mainly focussed on optimisation [11, 12], which assume the city as a blank page in which the infrastructure must be realised. These studies often rely on driving patterns from gasoline driven cars or geographical information such as income, urban density etc. in combination with assumptions about charging strategies and EV adoption [13, 14]. In reality, the realisation and expansion of infrastructure is much more complex, continuous operation in which local circumstances play a major role. Government incentives vary, which creates temporal dynamics in the EV adoption rate. Technological developments lead to new patterns in charging behaviour, which have been difficult to predict [15]. Charging point operators (CPOs) do not operate in a blank page, but continuously expand the current infrastructure in which they decide where and when to install the next station, instead of installing a multitude of chargers at once. From a CPO and policy maker perspective, a prioritisation is required, as funding and capacity of sufficiently qualified personnel are limiting factors. Strategies for expanding charging infrastructure in already existing networks have been missing so far.

This paper aims to fill this gap in two steps. First, the paper aims to define a measure of charging infrastructure success that copes with the multidimensionality of the problem at hand. To this end, the literature on charging infrastructure planning is reviewed with respect to performance indicators. Following this analysis, a new multi-dimensional standard for charging infrastructure success is proposed. Secondly, the paper develops a model to identify the variables that influence the success of charging infrastructure in existing networks with the use of data from the use of charging stations. This is done by using a large database with 1708 charging stations and 8.5 million charging sessions recorded from 2016 to 2020 in the Netherlands.

## 2. Literature review on electric vehicle charging station performance

A large part of the literature on electric vehicle charging infrastructure focuses on charging infrastructure planning. Reviews on the literature on this topic [16–18] show that the planning studies can be divided into node [19], tour [20] and path-based studies. Most studies, although not all, assume the city (or country) as a blank page in which charging infrastructure should be realised. Charging infrastructure is optimised according to a different set of parameters or optimisation.

The work from Motoaki shows that such planning studies however often do not resemble practice as local circumstances and policies play a major role in investment decisions as well as the historic development of charging infrastructure roll-out. Other studies take a broader look by not simply looking at actual locations but include business decisions [21–23] or look at stakeholder interests in general [24]. More recent work uses initial roll-out of charging infrastructure in frontrunner cities as a proxy to estimate infrastructure demand [20]. These studies show that geographic information systems (GIS) can be used for planning by using factors such as the built environment and income at the neighbourhood level [12, 25]. Adenaw & Lienkamp [26] use these GIS models, which are now widely used in practice, as an input into a 4-step process which agent-based modelling and a heuristic-based approach for location decisions to match more commonly used practices.

Charging station performance is used as a condition in charging station planning studies [14, 27]. Performance at the charging station level is measured as energy transferred to match requirements of a positive business case for the charging station owner [22, 28, 29]. Charging stations success is however secondary to the broader goal of meeting transportation needs from EV drivers in most of these studies. Gnann, Plötz, et al., for example try to minimize the number of charging stations to facilitate all charging sessions but include in their model that charging stations must be profitable for them to be actively operated. Islam et al. [30] try to minimize detour times for drivers using public data from travel times from Google maps. Other studies that focus on the charging point operator side also focus on the business case of charging points, trying to calculate the turnover needed in different scenarios or tariff settings [31–33]. These latter studies have a purely economic approach to the matter, while measuring performance could benefit from a broader perspective.

A similar trend is seen in descriptive studies on charging infrastructure use or charging behaviour. The focus is on the electric driver, partly because the data collected is from a drivers’ perspective [34–36] but also studies that collect data at the charging point translate this into the user behaviour [37–39] Other studies do focus on the charging point perspective such as Morissey, Weldon & Mahony [40] the heterogeneity in charging needs across different types of infrastructure. Gnann et al. [41] combine data from fast charging stations with travel data to develop a queuing model. They include the user perspective by examining the average queuing time but also include charging station metrics such as occupancy and profitability of the charging station. The vantage point for charging station success varies between EV drivers and the CPO across the different studies.

Charging station success is discussed in most studies regarding charging infrastructure but has so far been the focus point in only a limited number. Work by Helmus et al. [42] focusses on comparing success for different roll-out strategies among multiple performance indicators such as occupation, unique users and charging time. Helmus & van den Hoed [43] looked at a wide array of performance indicator and discussed their implications for policy makers. Straka et al. [44] tried to predict charging station success based on contextual factors. Success was defined with seven different indicators such energy consumed and the number of different cars charging at a charging station. Similar work was done by Almaghrebi et al. [45] which compared different machine learning techniques to predict success. Success was defined one-dimensional in terms of energy drawn from the charging station. A general definition of success has been missing so far in literature and has not been a specific focus point of research.

### 2.1 Contributions

The review on charging infrastructure performance has shown that current research on charging station performance has been limited and that an analysis of how current charging station performance influences future performance is missing. As well the review has shown that roll-out simulation studies that include charging station performance as a metric, mainly focus on the business case of the charging station and therefore the energy drawn from the station. The multidimensionality of the problem which many policy makers and charging point operators face has not been translated in the relevant Key Performance Indicators (KPIs) in literature.

This paper aims to fill the research gaps as identified as above. A systematic analysis of charging station performance indicators is proposed based upon a literature review and a reflection on the pros and cons of these indicators is given in the *section Key performance indicators for charging stations (Section 2)*. This results in a proposal on how to systematically evaluate charging station performance. In the section *Predicting charging station success (Section 3)*, this paper aims to provide insight into the relationship between current charging station network performance and the performance of newly installed stations. This contributes to the body of knowledge on how to expand charging station networks while many studies have focused on building a network from scratch.

## 3. Key performance indicators for charging stations

### 3.1 Methodology to determine KPIs

**Fig 1:**
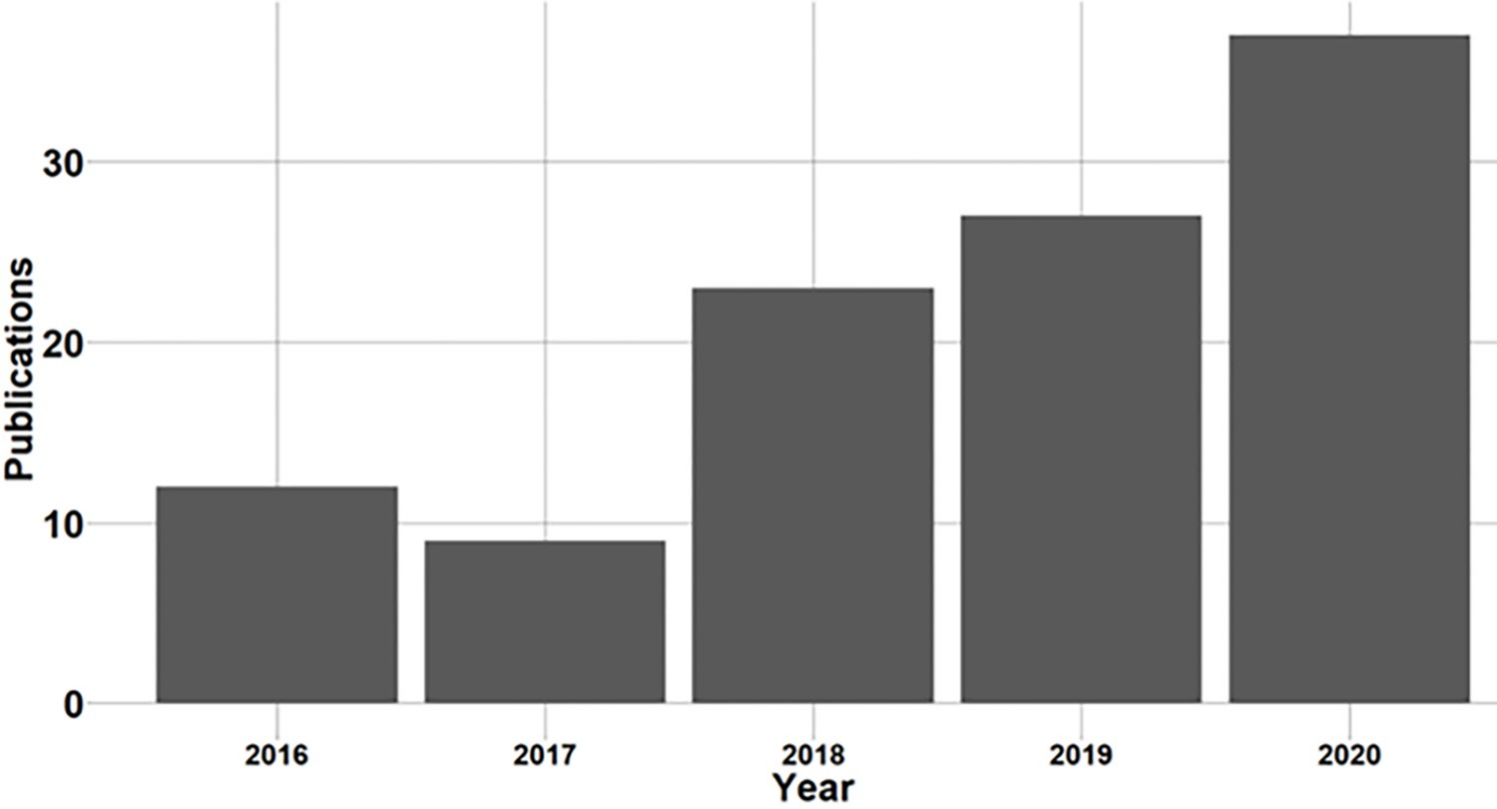
Number of publications for over time.

Literature on charging station deployment and performance is used to derive the most used KPIs. Literature was found using *Web of Science* with the key words *Electric vehicle charging infrastructure* in the title of the paper. The search focussed on papers from 2016 to 2020 to only include more recent papers. This choice was made as a rapid development in electric vehicle adoption and strategies for charging station placement has taken place during this time. This resulted in 108 papers. An overview of the number of papers in the database over time is given in Fig 1, which shows a clear increase in the number of papers published over the years.

To be able to make a sound review of the KPIs used in this paper only the top 25 cited papers were used for this analysis. Citations were reviewed as average citations per year to prevent a bias towards older papers. Abstracts were reviewed to see if these papers were about charging infrastructure planning or performance. This review resulted in omitting papers which sole focus was on shared or autonomous vehicles or purely focussed on electricity grid integration. The 25 papers that were reviewed can be found in Appendix A in S1 Appendix. Several papers also included KPIs that focussed on different actors such as the EV driver or the grid operator. We have excluded these KPIs from this analysis to only focus on KPIs that are related to charging infrastructure performance. The number of papers was limited to 25, to make a thorough review of these papers. Also, it was noted during the evaluation that after the initial top 15 papers, no new KPIs were found.

### 3.2 Results

The results of the analysis (Table 1) shows that ‘Energy (kWh)’ is seen as the most used performance indicator for charging infrastructure, highly related with the turnover at these charging stations. Turnover is however sometimes explicitly mentioned as a different indicator as this could depend on the tariff system used at these stations or from different sources such as advertisement. The energy drawn is closely related to the power of the charging stations at a specific time. Although papers exclusively aimed at grid integration were excluded from the analysis, this topic has a high priority among researchers in this field. Peak power, or ways to reduce this, are a prominent indicator for charging station performance.

**Table 1:** Performance indicator use in top 25 scientific studies on EV charging infrastructure.

Performance indicator	Used in # studies
Energy (kWh)	12
Sum of time connected/charging to charging station (hours)	9
Power (kW)	8
Turnover (€/$ etc.)	6
Occupancy rate (%)	5
No. of charging sessions	4
Social costs	3
Turnover from advertisement (€/$ etc.)	2
Unique users	1

The second most mentioned performance indicator is the time connected or charging at a station. These sometimes overlap, especially in case of DC fast charging stations, but are also sometimes explicitly different in case of slower charging at AC charging stations. EV drivers park at these charging stations and time connected leads to unutilised connection time. A different measure related to connection time often mentioned is the occupancy rate displayed as % of the time connected. The studies relate this occupancy rate to parking issues as fears over underutilisation compared to regular parking spots is an issue for policy makers.

Broader indicators for charging station success include the number of charging sessions and unique users at these stations. The number of charging sessions and unique users are different KPIs for serving the demand from EV drivers. In different areas these can have different implications. A high number of different users might indicate an area with a lot of visitors, which could in turn lead to a lot of charging sessions. Locations outside these areas might have a few frequent users but still have a similar or even higher number of charging sessions. Social costs parameter studies try to include aspects such as impact on the local environment and equality issues, but its application varies across the papers.

Based on this analysis, this paper focuses on four KPIs of EV charging which cover the broad aspects of EV charging. These are: Energy (kWh), time connected to the charging station, number of charging sessions and number of unique users. The factor of turnover is left out as this highly correlates with energy drawn, assuming a per kWh/charge or a time connected charge. Analysing this separately would not yield new results. Similarly, occupancy rate is not included as it is a different way of measuring time connected to a charging station. The KPI of power is limited due to the nature of the charging stations analysed in this aspect, maximum 11kW. Social costs were found to be varying in definition across the papers and therefore not suitable in this analysis. Turnover from advertisement is not related to roll-out strategy and given the diverse nature of this income any analysis would not lead to generalisable conclusions. This leaves four relevant KPI’s to be included in this research.

## 4. Predicting charging station success

### 4.1 Approach

**Fig 2:**
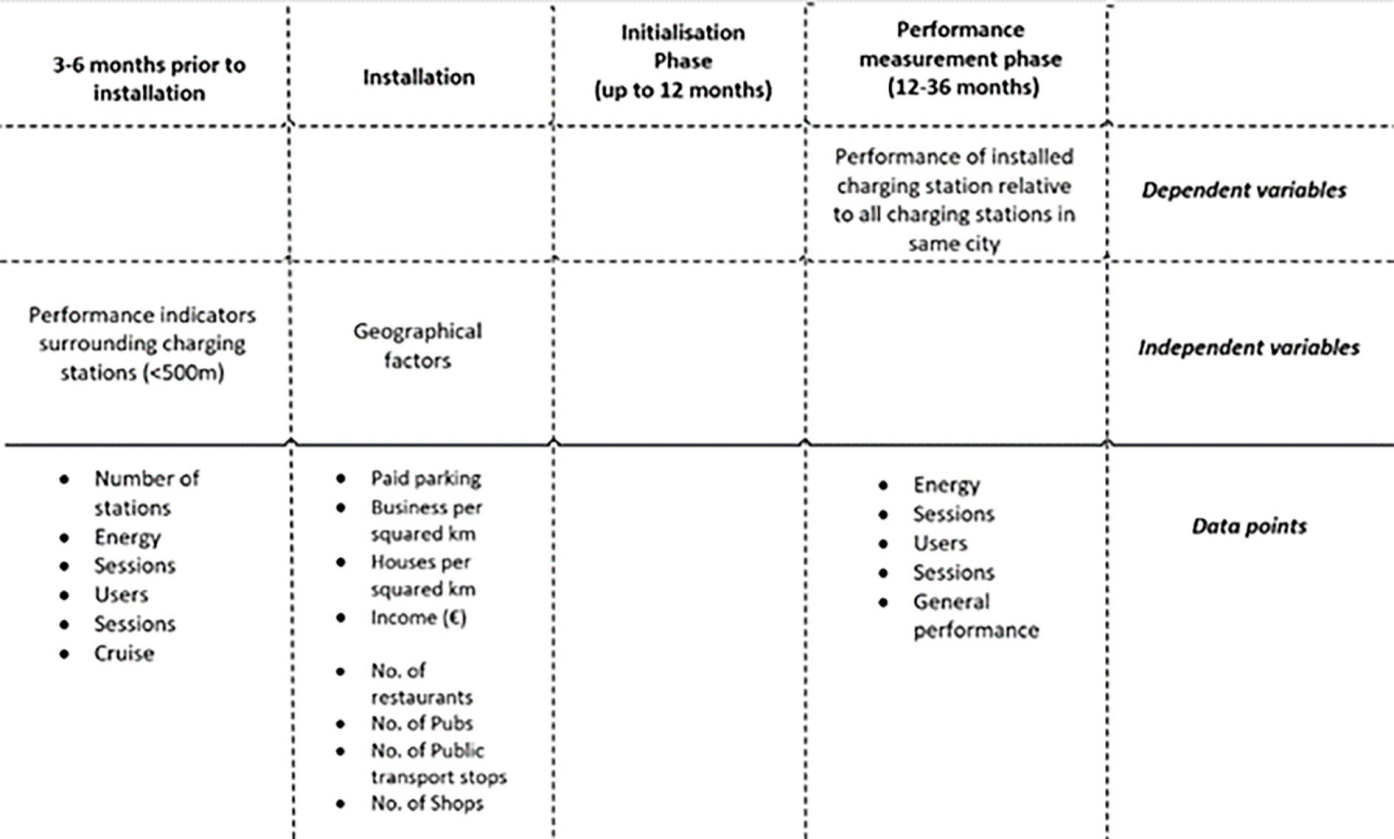
Conceptualisation of approach including timeline and obtained data points.

The approach proposed in this paper builds upon the idea that the success of newly placed charging stations depends on the performance of charging stations in the surroundings in the time leading up to the placement of this newly placed station. This would allow CPOs to assess the placement of new charging stations not only based upon parameters of the built and social environment, on which most models rely, but also incorporate actual charging network performance in their decision-making process. This concept would better fit with the daily operation and prioritisation within the decision-making process of charging stations. This idea and timeline of measurement has been visualised in Fig 2.

To assess which charging stations perform well, the four Key performance indicators for charging stations are used and an additional performance indicator for general success. This overall performance indicator encompasses the four performance indicators as one (see section 4.2 on the calculation).

To predict which charging stations are a success the same four KPIs of the surrounding charging stations are used with an addition of a social network performance indicator, which allows to not only view the performance of individual charging stations in the surrounding of the newly placed charging station, but also the social network effects between them. This is measured by the number of users that must deviate from their favourite charging station (close to home or workplace) to one in the surrounding. This behaviour indicates that charging station have more demand than station can handle and therefore users must search for other available stations. This cannot be directly measured by the four proposed KPIs and therefore this new parameter is created.

### 4.2 Methodology predicting success

To predict charging station success data from public charging stations from four Dutch cities are used (Amsterdam, Rotterdam, Utrecht, The Hague). Data from level 2 charging stations (2 sockets each up to 11kW) were collected from 2014 up to and including February 2020. Collection of the data was done through a collaboration with CPOs that operate in these cities within the Future Charging research program. Data collection methods have varied throughout the years and depending on the CPO. Most recently, data is being collected through a direct connection with OCPI protocol. Data is stored in a MS SQL database. Data beyond February 2020 was also collected but not considered due to the impact of the Covid-19 lockdown. Data from 1708 charging stations and 8.5 million charging sessions were collected. Data as in Table 2 were collected for each charging session. More on the data collection, processing and storage etc. can be found in Wolbertus et al. [46].

**Table 2:** Collected data.

Data	Unit
Location	Coordinates (Latitude, Longitude)
ChargePoint ID	Unique identifier for charge point
RFID ID	Unique identifier for EV driver
Start Time	Date and time of day
End Time	Date and time of day
Energy charged	kWh

To measure charging station success data was transformed to create a single datapoint per KPI per charging station each week of which in the average was taken to create a single datapoint per charging station (1708 in total). The timeframe of a week is used as it is consistent in length (compared to months) and has a regular pattern in terms of work and weekend days. KPIs are a sum of the number of kWh charged, unique users, time connected and number of charging sessions (see section 3.2 for the selection of these KPIs). To compare success, the KPIs are normalised in comparison to charging stations within the same city in the same week. Data is normalised by dividing the KPI by the average of all charging stations in the same city in that respective week. Hence, the score will be 1 if the charging station performs exactly the average. As an example, if at the charging station 100 kWh is charged in week 1 and the average across all charging station in week 1 is 200 kWh, the score is 0.5 (for easier interpretation this is later multiplied with 100). The average of this score across 2 years (12–36 months after installation) is used in the model, this results in a single data point per station. This comparison overcomes differences in adoption levels over time. Data normalisation allows to combine the four KPIs into a single parameter for charging station success. A fifth KPI is therefore calculated that is again normalised after normalised scores from each KPI are averaged. This fifth parameter (average score) is the general performance of charging station. Data is also reviewed considering the age of the charging station. The placement date is considered the first time a charging station is used, as charging stations are tested on site after installation. A single data point, the average performance per week for each charging station is entered into the model. This is the average of the weeks in year 2 and 3 after the installation (see section 4.3 on why only these weeks are considered in the model). In total 1708 charging stations were included in the analysis. Table 3 provides and overview of the dependent variables.

**Table 3:** Overview of dependent variables.

Dependent variables	Description	Measure
*Energy*	Amount of kWh	kWh compared to average in week of datapoint
*Number of Users*	Unique number of users measured by RFID-tag	n users compared to average in week of datapoint
*Connection Time*	Time connected (charging and not charging) to the station	Total connection time compared to average in week of datapoint
*Number of Sessions*	Number of charging sessions in given week	n sessions compared to average in week of datapoint
*Overall score*	Average performance across four KPIs above	Sum of average scores

Charging station success is modelled using a linear regression model on each of the KPIs (Model specification is given in Appendix D in S1 Appendix). Predictor variables include a summary of several statistics from surrounding charging stations that were there prior to the installation of the new charging station. Charging stations within 500 meters (as-the-crow-flies distance) were selected. Different radiuses were tried, but either resulted in a lack of data or no significant differences between the models could be found. Data was collected from six to up to three months prior to the installation of the charging station. This period was selected in consultation with policy makers and charging station operators. The period resembles the real-world decision period for expansion of charging networks. Three months are needed on average to arrange permits, grid connection and placement of the charging station. A three-month observation period was chosen to prevent incidental outliers in the data to inform decisions.

Predictor variables include number of sessions, energy charged (kWh), unique users, time connected (hours) and the number of charging stations in the given radius. Additionally, a variable is added to express the convenience in finding a charging station (see section 4.1 for an elaborate explanation). This is expressed as the share of charging sessions that do not take place at favourite charging station of the EV driver. The favourite charging station is determined by selecting the most used charging station of the EV driver which is identified by its RFID ID. Only EV drivers that charge at least 5 times in the same area are taken into consideration, as the favourite charging station cannot be determined for visitors.

To control for effects of the area in which the charging station is placed, geographical information on the sub-district level on income, type of built environment and paid parking is added [47]. Information about the facilities in the surrounding are retrieved from the OSRM database using the nominatim package in R. Given that charging stations provide a maximum of 11kW these stations are mostly used for charging while being parked for a longer time. The research focusses on facilities that would allow for longer parking times and not necessarily attract traffic for stop & go charging (e.g. fast charging > 50kW). The number of facilities is counted within a 500-meter range (as-the-crow-flies distance) of each charging station. Other distances (see Appendix B in S1 Appendix) were tried as well, but 500 meters provided the best fit. Data was retrieved only once (2020) and has been kept constant for the charging stations across time. A dummy variable is added to indicate the year in which the charging station was first used, 2014 is the reference level. Controlling for these factors also gives the opportunity to compare the model based on charging data to those models that solely rely on geographical information. The added value of including current charging stations performance can be estimated. Table 4 provides an overview of all independent variables used.

**Table 4:** Overview of independent variables.

*Variables*	Description	Measure
*Control variables*		
**Year [2015]** (Ref. 2014)	Year of instalment charging station	0 or 1
	Dummy for the year 2015– reference 2014	
**Year [2016]** (Ref. 2014)	Year of instalment charging station	0 or 1
	Dummy for the year 2016– reference 2014	
**Year [2017]** (Ref. 2014)	Year of instalment charging station	0 or 1
	Dummy for the year 2017– reference 2014	
**Year [2018]** (Ref. 2014)	Year of instalment charging station	0 or 1
	Dummy for the year 2018– reference 2014	
**Year [2019]** (Ref. 2014)	Year of instalment charging station	0 or 1
	Dummy for the year 2010– reference 2014	
*Variables related to charging stations <500m in 3–6 months prior to installation*		
**Number of stations**	Number of charging stations in the surrounding (< 500m)	Count
**Number of sessions**	Average number of charging sessions per station for charging stations in the surrounding (< 500m)	n sessions
**Number of users**	Average number of users per station for charging stations in the surrounding (< 500m)	No users
**Connection Time**	Average sum of time connecte per station for charging stations in the surrounding (< 500m)	hours
**Energy (kWh)**	Average energy charged per station for charging stations in the surrounding (< 500m)	kWh
**Share of sessions not at favourite charging station**	% of sessions not at favourite charging stations for frequent users (>5 sessions)	%
*Geographical factors (fixed over time)*		
**Paid parking**	Dummy for paid parking or not	0 or 1
**Business per squared km**	Share of buildings in the subdistrict assigned as business location	0 to 1
**Houses per squared km**	Share of buildings in the subdistrict assigned as housing	0 to 1
**Income (€)**	Average income in the subdistrict of the charging station	€
**No of restaurants (<500m)**	Count of number of restaurants in surrounding (<500m) of charging station	Count
**No of Pubs (<500m)**	Count of number of pubs in surrounding (<500m) of charging station	Count
**No of Public transport stops(<500m)**	Count of number of public transport stops (bus, tram, train) in surrounding (<500m) of charging station	Count
**No of Shops (<500m)**	Count of number of shops in surrounding (<500m) of charging station	Count

**Fig 3:**
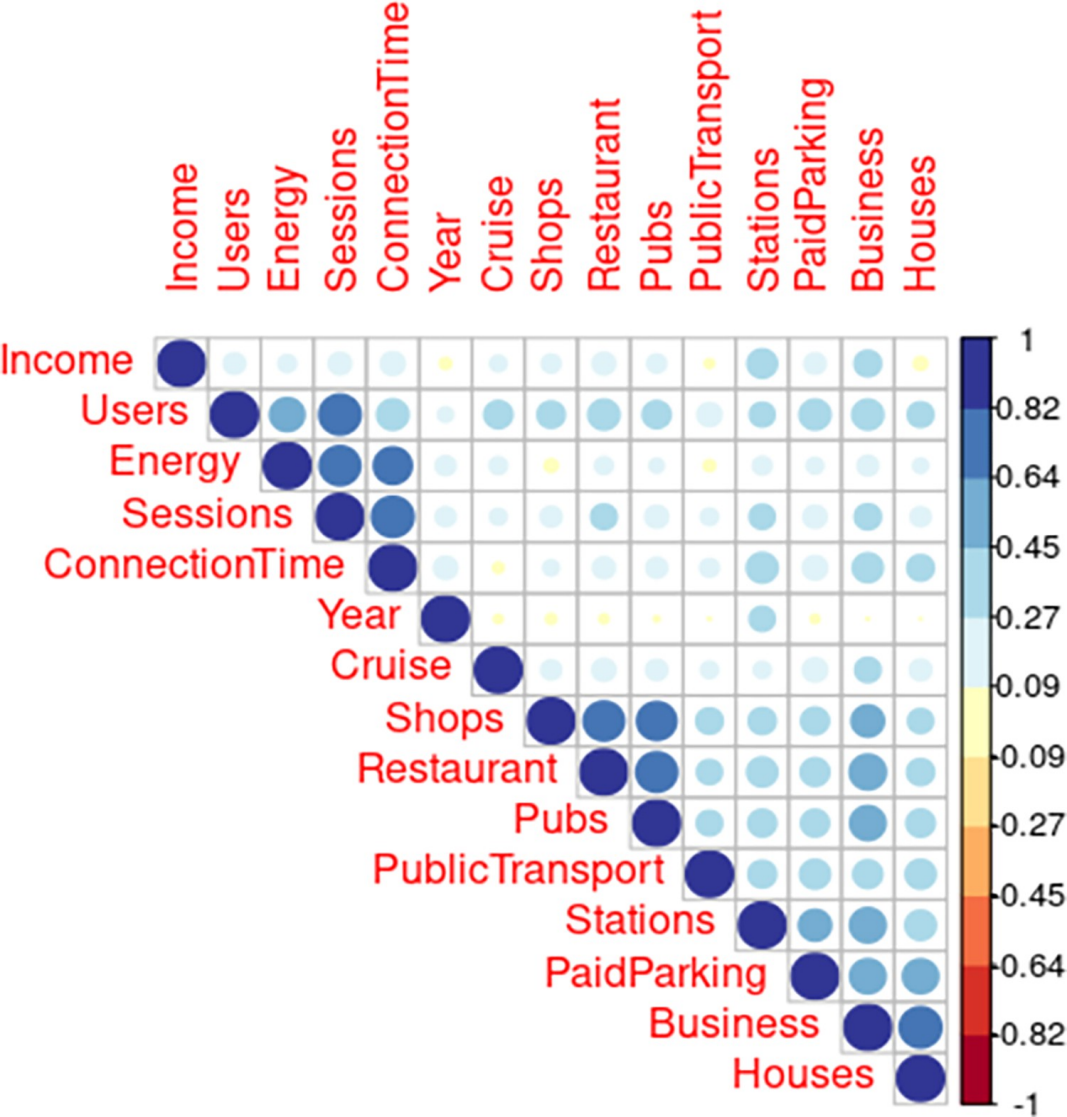
Correlation table.

The model was tested for multicollinearity using VIF scores. There was a significant correlation between number of sessions and other charging station related predictor variables (ranges between 0.7–0.8, see Fig 3, Appendix C in S1 Appendix). Yet, as these variables are central to the research question at hand these were not discarded. To test the impact on the results, the same model was tested with a stepwise regression. The stepwise regression results kept including all the different charging station variables across all models and therefore it was decided to include in the final model. To test for heteroskedasticities the Breugsch-Pagan test was used. Results were insignificant with exception for the model of connection time. A model on the log results of connection time were tested but this did not solve the heteroscedasticity. Given the similar results across all models, results are still considered useful. Results for multicollinearity and heteroskedasticities for each model can be found in Appendix C in S1 Appendix. Although no correlation of the error-terms was expected a Durbin-Watson test was performed for each model. Results (p = 0.4791) showed no correlation.

### 4.3 Descriptive results

Table 5 has descriptive statistics for the charging data used in this research across all years. It shows that on average nearly 10 kWh is charged per session, amounting to 117 kWh per charging station (2 points) in 12 charging sessions per week. These charging sessions are done by more than 6 different users per station each week, although there is a large variability in this number. Average power is below 1kW far below the rated power (3.7-11kW) of the stations indicating sufficient idle time during the charging sessions.

**Table 5:** Descriptive statistics for charging data.

KPI	Per charging session	Per week
**Energy**	9.7 kWh	117. 0 kWh
**Time connected**	10.3 hours	124.2 Hours
**Number of sessions**	Not applicable	12.0 sessions
**Number of unique users**	Not applicable	6.6 users

**Fig 4:**
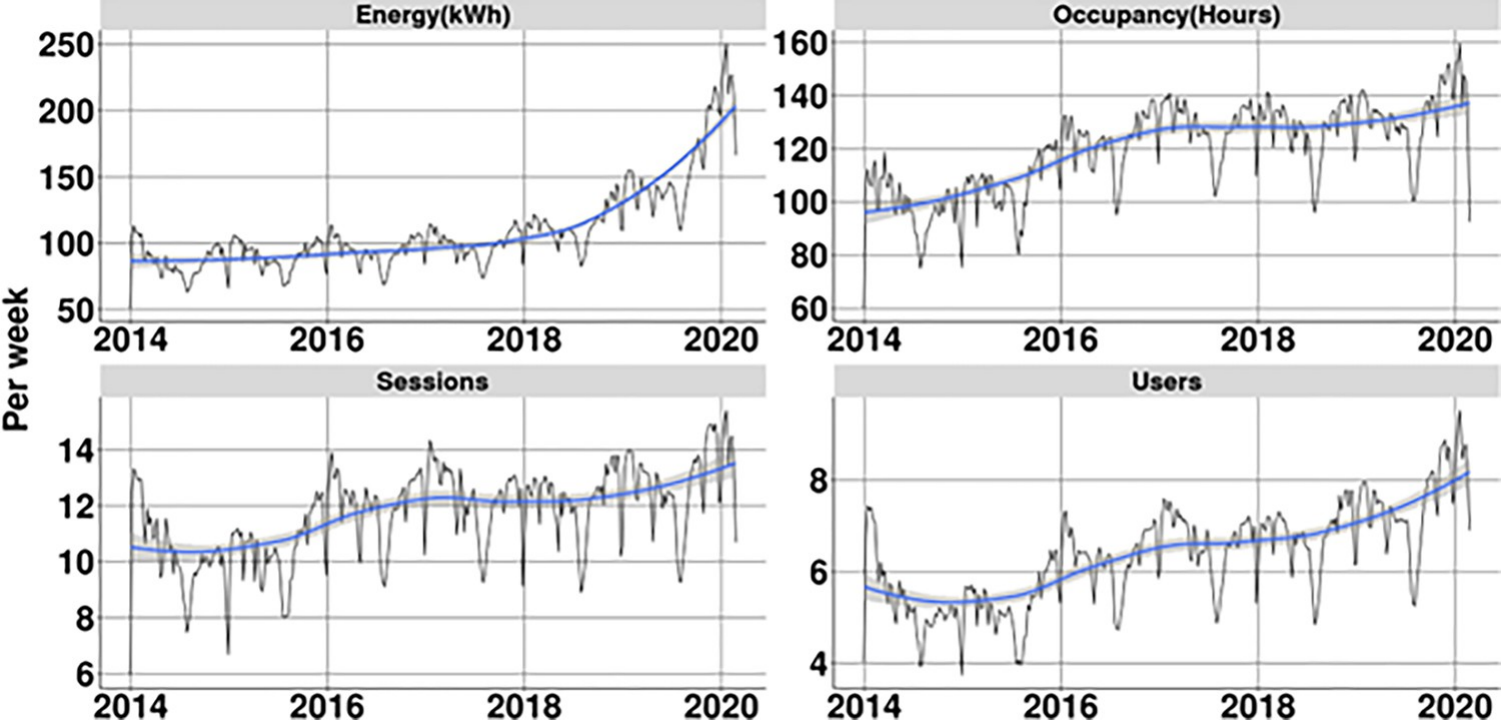
Development of key performance indicators for charging infrastructure over time. Black line shows actual measurement, blue line shows rolling average. The analysis therefore focuses on the performance of charging stations relative to each other in the same week of time, but also takes into account the age of the charging station. The performance relative to the age (compared to charging stations in the same week) is shown in Fig 5. This figure shows that for all four considered KPIs that newly placed charging stations take up to a year to perform at the same level as other charging stations within the same city. Normalisation compared to the age of the station shows that the KPIs all follow a similar pattern which allows them to be included in a general overall indicator. In the regression model only performance in year two and three after placement of the charging station is therefore considered. Beyond this the number of charging stations drops significantly as most stations were placed in the period 2017–2018. Analysis of such figures is therefore no longer considered reliable.

Fig 4 shows the development of the KPIs over time given per week. This shows a strong growth for most KPIs. This corresponds both the increased adoption of EVs in the Netherlands as well the shift from plug-in hybrid vehicles to full electric, due to a change in government incentives [48]. This increased the average energy per charging session dramatically, from 8.4 kWh in 2014 to 15.8 kWh in early 2020. All other factors show a smaller increase, indicating increased utilisation of the charging stations as a result of a larger number of EVs on the road. These are however limited in their increase due to the rival nature of charging stations; once occupied they cannot be used by others.

**Fig 5:**
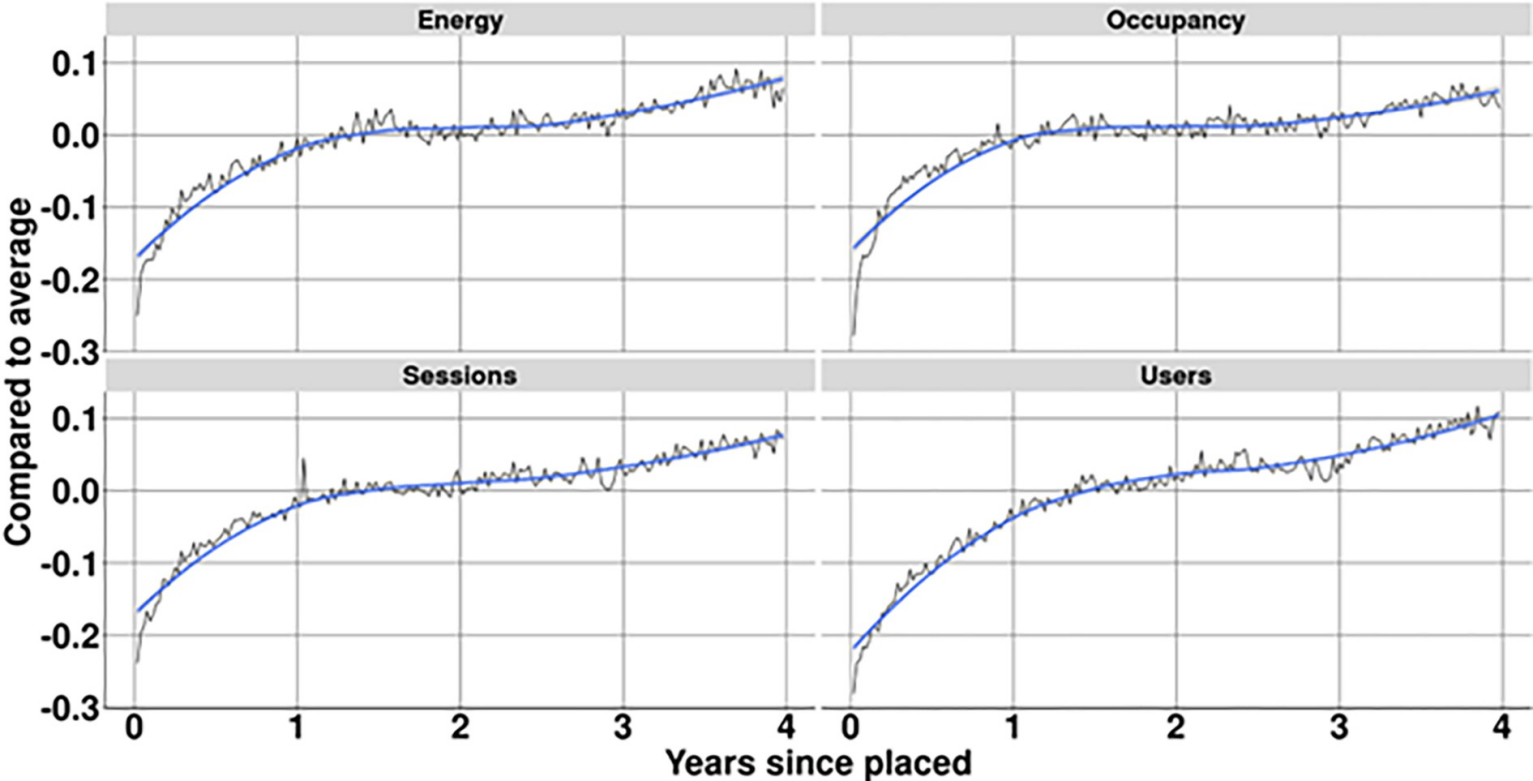
Development of key performance indicators compared to mean performance in the same city in relation to age. Black line shows actual measurement, blue line shows rolling average.

### 4.4 Model

Regression models are estimated for all different KPIs and results for all shown in Table 6. R^2^ is displayed for each model, and p-values for each variable. Bold font indicates significance at 0.05 level for each of the variables. The predictor variables are split into three categories, intercept and dummy variables for year of charging station placement, variables related to the surrounding charging stations and geographical factors.

**Table 6:** Model results.

	Average score		Energy (kWh)		Connection Time (Hours)		Unique users		Number of sessions	
*Predictors*	*Beta Coefficient*	*p-value*	*Beta Coefficient*	*p-value*	*Beta Coefficient*	*p-value*	*Beta Coefficient*	*p-value*	*Beta Coefficient*	*p-value*
**Intercept**	65.52	**<0.001**	78.79	**<0.001**	64.93	**<0.001**	55.36	**<0.001**	63.00	**<0.001**
**Year [2015]** (Ref. 2014)	-9.76	**0.003**	-13.61	**<0.001**	-7.71	**0.047**	-12.49	**0.003**	-5.24	0.139
**Year [2016]** (Ref. 2014)	-13.40	**<0.001**	-19.83	**<0.001**	-13.29	**0.001**	-12.67	**0.003**	-7.83	**0.029**
**Year [2017]** (Ref. 2014)	-18.60	**<0.001**	-20.10	**<0.001**	-22.62	**<0.001**	-16.78	**0.001**	-14.89	**<0.001**
**Year [2018]** (Ref. 2014)	-18.13	**<0.001**	-22.40	**<0.001**	-21.25	**<0.001**	-17.24	**0.001**	-11.63	**0.006**
**Year [2019]** (Ref. 2014)	-18.63	**0.006**	-25.46	**0.002**	-24.86	**0.002**	-15.13	0.082	-9.07	0.218
*Variables related to charging stations <500m in 3–6 months prior to installation*										
**Number of stations**	0.70	**0.01**	0.35	0.361	1.74	**0.048**	0.27	0.520	0.42	0.229
**Number of sessions**	0.73	**0.01**	0.53	**0.007**	0.39	**0.001**	1.15	**<0.001**	0.86	**<0.001**
**Number of users**	-0.51	**-0.01**	-0.55	**0.028**	-0.82	**0.029**	-0.09	0.751	-0.59	**0.011**
**Connection Time**	-0.03	**-0.00**	-0.03	**0.015**	0.03	0.521	-0.07	**<0.001**	-0.03	**0.014**
**Energy**	0.01	**0.00**	0.04	**<0.001**	-0.01	**0.013**	-0.01	0.513	0.00	0.582
**Share of sessions not at favourite charging station**	14.13	0.14	13.06	0.052	16.56	**0.006**	6.98	0.332	19.93	**0.001**
*Geographical factors*										
**Paid parking**	15.54	**<0.001**	12.06	**<0.001**	8.78	**0.006**	30.34	**<0.001**	10.98	**<0.001**
**Business per squared km**	-0.41	**0.011**	-0.36	0.064	-0.19	0.321	-0.63	**0.002**	-0.48	**0.006**
**Houses per squared km**	0.05	0.323	0.02	0.732	0.11	0.075	0.03	0.661	0.05	0.410
**Income (€)**	0.36	**<0.001**	0.37	**<0.001**	0.30	**0.001**	0.43	**<0.001**	0.35	**<0.001**
**No of restaurants (<500m)**	0.30	**0.001**	0.11	0.282	0.10	0.325	0.73	**<0.001**	0.24	**0.009**
**No of Pubs (<500m)**	0.34	0.256	0.01	0.968	0.66	0.064	0.35	0.363	0.35	0.283
**No of Public transport stops (<500m)**	0.37	0.070	0.43	0.075	-0.24	0.328	0.83	**0.002**	0.47	**0.035**
**No of Shops (<500m)**	-0.02	0.531	-0.03	0.411	-0.05	0.144	0.00	0.901	0.00	0.977
**Observations**	1708									
**R** ^**2**^ ** **	0.186		0.096		0.146		0.303		0.128	

In general, the predictive value of the models is relatively low, varying from a R^2^-value of as low as 0.096 for the energy model to as high as 0.303 for the unique user model. Dummy variables for the year of installation are significant in all models, but the relative importance of these dummies is rather low (~5% of the explained variance). The dummy variables for year installed follow the trend as shown in Fig 4 that older charging stations perform better on all variables, hence all year-dummies are negative as they compare to the oldest stations installed in 2014. In general, it is found that variables related to the charging stations have the highest relative importance (45–55% of explained variance) followed by geographical factors (35–45% of explained variance).

### 4.5 Variables related to charging stations in the surrounding

As it comes to predictors of the measured performance of neighbouring charging stations it can be found that the number of charging sessions is significant parameter in all of the models. The number of sessions is also the parameter with the highest relative importance among the factors related to surrounding charging stations (13.3% of explained variance in the average score model and 19.4% in the model for sessions). The number of sessions is best predictor for charging station success for all relevant KPIs, implying that simply counting sessions is a good indicator for charging demand.

Current occupation of charging stations, measured in the connection time is a significant parameter in all models. The predictive value, is much less than parameter that represents the number of charging sessions. This is because in some parts of the cities charging stations are mainly used for overnight charging. This leads to high occupation but a lower score on other factors such as energy, number of sessions and unique users. Some of these charging stations are only used by a few frequent users which use the charging station as substitute for home or office charging which results in high occupation but lower scores for other KPIs. A similar trend is seen for the energy parameter, results are significant, but the relative importance is very low. This can be explained by the fact that the energy parameter input is also highly dependent on the battery size of frequent users of the charging stations. Frequent plug-in hybrid users could lead to a high number of sessions and high occupation but low energy, compared to a full electric vehicle with a large battery. A newly installed charging station is not likely to attract the frequent users of already existing stations, and the battery size of new users is hard to predict. This is more likely dependent on government incentives for type of vehicles (e.g., PHEV vs FEV subsidies).

Remarkable is the finding that a high number of unique users leads to lower performance across all parameters (except the number of users). The interpretation is that a high number of unique users indicates a place suitable for a lot of infrequent visitors to city (e.g., for shopping, commercial visits, tourists). A similar trend is observed in the No. of businesses parameter. These stations do not have frequent users which perform better on the other KPIs as they often make daily use of the charging stations. Infrequent users are also likely to only stay for a shorter time (impact on connection time and energy).

The share of charging sessions not at the favourite charging station is a significant factor in 2 out of 5 models. Current users cruising to find an available charging station are a relevant predictor for charging station success in the future. Remarkable is that the current performance in terms of energy, and thus business case, is only significant in the energy model itself and has a high relative importance in this model as well (18.4% of variance explained). This also indicates that users from the same neighbourhood will make use of new charging stations in the surroundings, leading to a higher turnover. The actual number of stations within the 500-meter radius is a significant parameter in most models, but its relative importance is low, explaining only a few percent of the variance in each of the models.

### 4.6 Geographical factors

The geographical variables have a significant contribution to the model’s performance showing that a combination of current performance and geographical based factors can complement each other in predictions of charging station success. This is relevant for all models with a relative importance varying between 27% (Number of sessions) and 43% (Unique users) across the different predicted KPIs.

The models show that paid parking and the income level in a neighbourhood are the best predictors for charging station success. Especially paid parking is a good predictor for the number of different unique users, in line with expectations. Paid parking areas are mostly close to the city centre and therefore will attract different EV drivers that do not charge their EV regularly in the area. In the model used to predict the number of unique users, this is parameter accounts for 27.4% of the explained variance. However, it is not only a good predictor for the number of unique users, but also for other factors such as energy which was less in line with expectations. This could be explained by the fact that these users have relative short charging sessions in which most of the time is used for actual charging instead of idle connection time.

The income level is also a highly significant parameter in most models. Income levels are known to be a good predictor for EV adoption as purchase prices of these models are higher than gasoline equivalents. As well, higher income levels are more likely to purchase or lease new cars than relative lower income levels. Yet, it is striking that this also results in a better charging station performance, as the number of charging stations should grow alongside the number of EVs. Possible explanation is that in neighbourhoods with higher income levels a larger network of charging stations is present. This leads to network effects in which charging stations are better used as a whole, while the EV driver does not experience a reduced availability.

Housing and business density have little predictive value for charging station success. In general, the parameters could indicate general charging demand, but this does not influence charging station performance, or this is captured by the income variables. Only the number of businesses is a significant parameter for the number of unique users as it indicates, just as paid parking, city centres with a lot of visitors.

More specific parameters on the neighbourhood facilities such as the number of shops, restaurants show that these can be of some influence but are often insignificant. Only the number of restaurants and number of public transport stops seems to have a small positive significant impact. The number of public transports stops has the highest impact of the number of unique users which has a relation with shared free floating electric cars which are seen as an extension of public transport. Shared electric cars are more likely to be charged near public transport stops. Given that free floating shared vehicle operators have a large fleet, of which the users can drive and park the car anywhere in the city, this could generate a lot of different users (as measured by looking at the ID of the car) at a charging station. The general lack of significance of these parameters could be explained by the idea that most public charging stations have mostly been placed to accommodate the inhabitants or visiting workers in mind. This than also presents most charging at these stations. The facilities included in this model would mainly be related to visitors in the city, which represent a fraction of the charging sessions and thus does not influence the performance of these stations.

## 5. Conclusions

Charging station performance is a multi-dimensional problem from a policy maker perspective. And should therefore be defined as such. This research proposes to analyse and predict success alongside four factors: energy, charging sessions, occupation and unique users. These factors should be compared to the performance of stations which operate in the same time span as the temporal aspects, due for example shifting incentives and battery developments at OEMs, are key factors that determine this performance. The proposed factors are also currently used in different ways to assess charging station performance in literature, although a structured approach to measuring success was missing.

With the use of the relevant KPIs this paper has assessed which factors could help predict charging station performance of new charging stations in current networks. Such a methodology is an addition to current models that predict EV uptake based on geographical based models and would allow CPOs and policy makers to prioritise resources on a relative short term. Results show that indicators of current charging station performance add to the prediction power of geographical-based models, although general predictability of charging station performance is relatively low (maximum R^2^ of 0.259).

The number of charging sessions, occupation and share of charging sessions at non-favourite locations were found to the best predictors of charging stations success in a broad sense. In terms of business case, related to energy sold, the models showed poor predictability. Analysis of the same data as used in this paper showed that most charging stations are used by a limited number of frequent users [49]. The charging behaviour and battery capacity of those EV drivers have been found to highly correlate with performance in terms of energy charged. A charging station mostly used by two plug-in hybrid vehicles compared to two full electric vehicles will perform less in that respect but will show good performance in terms of occupation and charging sessions. This type of model is however not able to predict what type of vehicle new EV drivers purchase. This is however a dominant factor, especially in this case as the Netherlands switched incentives for PHEVs and BEVs radically over time. The model should therefore be expanded with a parameter that includes market segmentation.

### 5.1 Limitations

This paper is used data from charging stations in the Netherlands to calculate the impact of past charging station performance on future performance. The data that is used comes from dense urban areas in which many users rely on on-street parking facilities. As such, this research should also be interpreted on a case study for similar urban areas in which cities are asked to (co-)develop public infrastructure which elsewhere would be resolved by private and workplace charging.

The performance of charging stations, as shown by the results, is also very time dependent. Outside factors such incentives for placement and purchase subsidies can play a significant role. This research has included factors to account for such factors but not all variance is likely to be captured. Any application of these results should always be interpreted in the context of local circumstances. This research should be used to give indication on which factors, in which direction and the approximate size of the effect of these factors.

### 5.2 Policy and practical implications

Results show that a combination of geographical and current network performance complement each other in terms of forecasting charging station performance. As a result, policy makers and charge point operators would have to look for best practices to combine these. A recommendation would be to use geographical parameters in long term planning as these are best be suited to predict EV adoption and therefore infrastructure need on a larger scale. Based on this initial planning, prioritisation of resources is best done with use of the current network performance added with information on sales of electric vehicles in the neighbourhood. Frequent users still largely determine the performance of charging stations. However, if charge point operators look at performance on a more network level, information on network performance is highly relevant and placement strategies should also include network effects and not solely look at single point performance.

In general, this research shows that due to the high variability among the use of charging stations, the dominance of the type of user on level 2 charging infrastructure performance (due to the rival nature of a charging station) and variations in purchase policies overtime, charging station performance is difficult to model with a high certainty at the level of a single charging station. Future work could have a closer look at the influence of network size on the relevance of each indicator although interactions between these variables did not lead to increased performance of the models at hand. As well, due to low predictability at a single station level, further work could be done on the performance of the network as a whole or in certain neighbourhoods. This would moderate some of the effects found at the single station level.

## Supporting information

S1 Data The data file contains information about each charging location used to build the models. It contains information about the charging station performance and the explanatory variables that are used. Data can be used to recreate the model.
(XLSX)

S1 Appendix (DOCX)
