# Supplementary material for: Identifying factors that influence electric vehicle charging station performance in expanding networks
Source: PLoS One. 2024 Apr 26;19(4):e0302132. doi: 10.1371/journal.pone.0302132 (PMC11051612; doi:10.1371/journal.pone.0302132)
Supplement: S1 Appendix — (DOCX) [file pone.0302132.s002.docx]

**Appendix A**

| **Authors** | **Article Title** | **Source Title** | **Publication Year** |
| --- | --- | --- | --- |
| Das, HS; Rahman, MM; Li, S; Tan, CW  [1] | Electric vehicles standards, charging infrastructure, and impact on grid integration: A technological review | Renewable & sustainable energy reviews | 2020 |
| Hardman, S; Jenn, A; Tal, G; Axsen, J; Beard, G; Daina, N; Figenbaum, E; Jakobsson, N; Jochem, P; Kinnear, N; Plotz, P; Pontes, J; Refa, N; Sprei, F; Turrentine, T; Witkamp, B[2] | A review of consumer preferences of and interactions with electric vehicle charging infrastructure | Transportation research part d-transport and environment | 2018 |
| Rahman, I; Vasant, PM; Singh, BSM; Abdullah-Al-Wadud, M; Adnan, N [3] | Review of recent trends in optimization techniques for plug-in hybrid, and electric vehicle charging infrastructures | Renewable & sustainable energy reviews | 2016 |
| Gnann, T; Funke, S; Jakobsson, N; Plotz, P; Sprei, F; Bennehag, A [Cited in manuscript 41] | Fast charging infrastructure for electric vehicles: Today's situation and future needs | Transportation research part d-transport and environment | 2018 |
| Morrissey, P; Weldon, P; O'Mahony, M [Cited in manuscript 40] | Future standard and fast charging infrastructure planning: An analysis of electric vehicle charging behaviour | Energy policy | 2016 |
| Chen, ZB; Liu, W; Yin, YF[4] | Deployment of stationary and dynamic charging infrastructure for electric vehicles along traffic corridors | Transportation research part c-emerging technologies | 2017 |
| Greene, DL; Kontou, E; Borlaug, B; Brooker, A; Muratori, M  [5] | Public charging infrastructure for plug-in electric vehicles: What is it worth? | Transportation research part d-transport and environment | 2020 |
| Madina, C; Zamora, I; Zabala, E  [Cited in manuscript 33] | Methodology for assessing electric vehicle charging infrastructure business models | Energy policy | 2016 |
| Liu, JC; Wei, QS[6] | Risk evaluation of electric vehicle charging infrastructure public-private partnership projects in China using fuzzy TOPSIS | Journal of cleaner production | 2018 |
| Mehrjerdi, H; Hemmati, R[7] | Electric vehicle charging station with multilevel charging infrastructure and hybrid solar-battery-diesel generation incorporating comfort of drivers | Journal of energy storage | 2019 |
| Pagany, R; Camargo, LR; Dorner, W[Cited in manuscript 11] | A review of spatial localization methodologies for the electric vehicle charging infrastructure | International journal of sustainable transportation | 2019 |
| Fang, YJ; Wei, W; Mei, SW; Chen, LJ; Zhang, XM; Huang, SW [8] | Promoting electric vehicle charging infrastructure considering policy incentives and user preferences: An evolutionary game model in a small-world network | Journal of cleaner production | 2020 |
| Xie, F; Liu, CZ; Li, SY; Lin, ZH; Huang, YX[9] | Long-term strategic planning of inter-city fast charging infrastructure for battery electric vehicles | Transportation research part e-logistics and transportation review | 2018 |
| Davidov, S; Pantos, M[10] | Planning of electric vehicle infrastructure based on charging reliability and quality of service | Energy | 2017 |
| Lee, JH; Chakraborty, D; Hardman, SJ; Tal, G [11] | Exploring electric vehicle charging patterns: Mixed usage of charging infrastructure | Transportation research part d-transport and environment | 2020 |
| Ji, ZY; Huang, XL [12] | Plug-in electric vehicle charging infrastructure deployment of China towards 2020: Policies, methodologies, and challenges | Renewable & sustainable energy reviews | 2018 |
| Funke, SA; Sprei, F; Gnann, T; Plotz, P [13] | How much charging infrastructure do electric vehicles need? A review of the evidence and international comparison | Transportation research part d-transport and environment | 2019 |
| Chen, TJ; Zhang, XP; Wang, JJ; Li, JN; Wu, C; Hu, MZ; Bian, HP [14] | A Review on Electric Vehicle Charging Infrastructure Development in the UK | Journal of modern power systems and clean energy | 2020 |
| Lopez-Behar, D; Tran, M; Froese, T; Mayaud, JR; Herrera, OE; Merida, W [15] | Charging infrastructure for electric vehicles in Multi-Unit Residential Buildings: Mapping feedbacks and policy recommendations | Energy policy | 2019 |
| Ghamami, M; Zockaie, A; Nie, Y [16] | A general corridor model for designing plug-in electric vehicle charging infrastructure to support intercity travel | Transportation research part c-emerging technologies | 2016 |
| Zhang, LH; Zhao, ZL; Xin, H; Chai, JX; Wang, G[17] | Charge pricing model for electric vehicle charging infrastructure public-private partnership projects in China: A system dynamics analysis | Journal of cleaner production | 2018 |
| Micari, S; Polimeni, A; Napoli, G; Andaloro, L; Antonucci, V [18] | Electric vehicle charging infrastructure planning in a road network | Renewable & sustainable energy reviews | 2017 |
| Zhou, Y; Wen, RX; Wang, HW; Cai, H  [19] | Optimal battery electric vehicles range: A study considering heterogeneous travel patterns, charging behaviors, and access to charging infrastructure | Energy | 2020 |
| Pagani, M; Korosec, W; Chokani, N; Abhari, RS [20] | User behaviour and electric vehicle charging infrastructure: An agent-based model assessment | Applied energy | 2019 |
| Efthymiou, D; Chrysostomou, K; Morfoulaki, M; Aifantopoulou, G [21] | Electric vehicles charging infrastructure location: a genetic algorithm approach | European transport research review | 2017 |

**References appendix A:**

**[1] H. S. Das, M. M. Rahman, S. Li, and C. W. Tan, “Electric vehicles standards, charging infrastructure, and impact on grid integration: A technological review,” Renewable and Sustainable Energy Reviews, vol. 120, p. 109618, Mar. 2020, doi: 10.1016/j.rser.2019.109618.**

**[2] S. Hardman et al., “A review of consumer preferences of and interactions with electric vehicle charging infrastructure,” Transp Res D Transp Environ, vol. 62, pp. 508–523, Jul. 2018, doi: 10.1016/j.trd.2018.04.002.**

**[3] I. Rahman, P. M. Vasant, B. S. M. Singh, M. Abdullah-Al-Wadud, and N. Adnan, “Review of recent trends in optimization techniques for plug-in hybrid, and electric vehicle charging infrastructures,” Renewable and Sustainable Energy Reviews, vol. 58, pp. 1039–1047, May 2016, doi: 10.1016/j.rser.2015.12.353.**

**[4] Z. Chen, W. Liu, and Y. Yin, “Deployment of stationary and dynamic charging infrastructure for electric vehicles along traffic corridors,” Transp Res Part C Emerg Technol, vol. 77, pp. 185–206, Apr. 2017, doi: 10.1016/j.trc.2017.01.021.**

**[5] D. L. Greene, E. Kontou, B. Borlaug, A. Brooker, and M. Muratori, “Public charging infrastructure for plug-in electric vehicles: What is it worth?,” Transp Res D Transp Environ, vol. 78, p. 102182, Jan. 2020, doi: 10.1016/j.trd.2019.11.011.**

**[6] J. Liu and Q. Wei, “Risk evaluation of electric vehicle charging infrastructure public-private partnership projects in China using fuzzy TOPSIS,” J Clean Prod, vol. 189, pp. 211–222, Jul. 2018, doi: 10.1016/j.jclepro.2018.04.103.**

**[7] H. Mehrjerdi and R. Hemmati, “Electric vehicle charging station with multilevel charging infrastructure and hybrid solar-battery-diesel generation incorporating comfort of drivers,” J Energy Storage, vol. 26, p. 100924, Dec. 2019, doi: 10.1016/j.est.2019.100924.**

**[8] Y. Fang, W. Wei, S. Mei, L. Chen, X. Zhang, and S. Huang, “Promoting electric vehicle charging infrastructure considering policy incentives and user preferences: An evolutionary game model in a small-world network,” J Clean Prod, vol. 258, p. 120753, Jun. 2020, doi: 10.1016/j.jclepro.2020.120753.**

**[9] F. Xie, C. Liu, S. Li, Z. Lin, and Y. Huang, “Long-term strategic planning of inter-city fast charging infrastructure for battery electric vehicles,” Transp Res E Logist Transp Rev, vol. 109, pp. 261–276, Jan. 2018, doi: 10.1016/j.tre.2017.11.014.**

**[10] S. Davidov and M. Pantoš, “Planning of electric vehicle infrastructure based on charging reliability and quality of service,” Energy, vol. 118, pp. 1156–1167, Jan. 2017, doi: 10.1016/j.energy.2016.10.142.**

**[11] J. H. Lee, D. Chakraborty, S. J. Hardman, and G. Tal, “Exploring electric vehicle charging patterns: Mixed usage of charging infrastructure,” Transp Res D Transp Environ, vol. 79, p. 102249, Feb. 2020, doi: 10.1016/j.trd.2020.102249.**

**[12] Z. Ji and X. Huang, “Plug-in electric vehicle charging infrastructure deployment of China towards 2020: Policies, methodologies, and challenges,” Renewable and Sustainable Energy Reviews, vol. 90, pp. 710–727, Jul. 2018, doi: 10.1016/j.rser.2018.04.011.**

**[13] S. Á. Funke, F. Sprei, T. Gnann, and P. Plötz, “How much charging infrastructure do electric vehicles need? A review of the evidence and international comparison,” Transp Res D Transp Environ, vol. 77, pp. 224–242, Dec. 2019, doi: 10.1016/j.trd.2019.10.024.**

**[14] T. Chen et al., “A Review on Electric Vehicle Charging Infrastructure Development in the UK,” Journal of Modern Power Systems and Clean Energy, vol. 8, no. 2, pp. 193–205, 2020, doi: 10.35833/MPCE.2018.000374.**

**[15] D. Lopez-Behar, M. Tran, T. Froese, J. R. Mayaud, O. E. Herrera, and W. Merida, “Charging infrastructure for electric vehicles in Multi-Unit Residential Buildings: Mapping feedbacks and policy recommendations,” Energy Policy, vol. 126, pp. 444–451, Mar. 2019, doi: 10.1016/j.enpol.2018.10.030.**

**[16] M. Ghamami, A. Zockaie, and Y. (Marco) Nie, “A general corridor model for designing plug-in electric vehicle charging infrastructure to support intercity travel,” Transp Res Part C Emerg Technol, vol. 68, pp. 389–402, Jul. 2016, doi: 10.1016/j.trc.2016.04.016.**

**[17] L. Zhang, Z. Zhao, H. Xin, J. Chai, and G. Wang, “Charge pricing model for electric vehicle charging infrastructure public-private partnership projects in China: A system dynamics analysis,” J Clean Prod, vol. 199, pp. 321–333, Oct. 2018, doi: 10.1016/j.jclepro.2018.07.169.**

**[18] S. Micari, A. Polimeni, G. Napoli, L. Andaloro, and V. Antonucci, “Electric vehicle charging infrastructure planning in a road network,” Renewable and Sustainable Energy Reviews, vol. 80, pp. 98–108, Dec. 2017, doi: 10.1016/j.rser.2017.05.022.**

**[19] Y. Zhou, R. Wen, H. Wang, and H. Cai, “Optimal battery electric vehicles range: A study considering heterogeneous travel patterns, charging behaviors, and access to charging infrastructure,” Energy, vol. 197, p. 116945, Apr. 2020, doi: 10.1016/j.energy.2020.116945.**

**[20] M. Pagani, W. Korosec, N. Chokani, and R. S. Abhari, “User behaviour and electric vehicle charging infrastructure: An agent-based model assessment,” Appl Energy, vol. 254, p. 113680, Nov. 2019, doi: 10.1016/j.apenergy.2019.113680.**

**[21] D. Efthymiou, K. Chrysostomou, M. Morfoulaki, and G. Aifantopoulou, “Electric vehicles charging infrastructure location: a genetic algorithm approach,” European Transport Research Review, vol. 9, no. 2, p. 27, Jun. 2017, doi: 10.1007/s12544-017-0239-7.**

**Appendix B: Model results for different distances of facilities to measured charging station (200-300-400 meters)**

|  | **Average score (400m)** | | **Average score (300m)** | | **Average score (200m)** | |
| --- | --- | --- | --- | --- | --- | --- |
| *Predictors* | *Beta Coefficient* | *p-value* | *Beta Coefficient* | *p-value* | *Beta Coefficient* | *p-value* |
| **Intercept** | 65.79 | **<0.001** | 65.82 | **<0.001** | 67.51 | **<0.001** |
| **Year [2015]** (Ref. 2014) | -10.07 | **0.002** | -9.59 | **0.004** | -9.95 | **0.003** |
| **Year [2016]** (Ref. 2014) | -13.65 | **<0.001** | -13.25 | **<0.001** | -13.46 | **<0.001** |
| **Year [2017]** (Ref. 2014) | -18.89 | **<0.001** | -18.89 | **<0.001** | -19.22 | **<0.001** |
| **Year [2018]** (Ref. 2014) | -18.43 | **<0.001** | -18.36 | **<0.001** | -18.94 | **<0.001** |
| **Year [2019]** (Ref. 2014) | -19.32 | **0.005** | -19.17 | **0.005** | -20.21 | **0.003** |
| *Variables related to charging stations <500m in 3-6 months prior to installation* |  |  |  |  |  |  |
| **Number of stations** | 0.73 | **0.025** | 0.76 | **0.019** | 0.80 | **0.013** |
| **Number of sessions** | 0.78 | **<0.001** | 0.79 | **<0.001** | 0.76 | **<0.001** |
| **Number of users** | -0.51 | **0.017** | -0.48 | **0.025** | -0.41 | 0.055 |
| **Connection Time** | -0.03 | **0.021** | -0.03 | **0.018** | -0.03 | **0.028** |
| **Energy** | 0.01 | 0.360 | 0.01 | 0.404 | 0.01 | 0.469 |
| **Share of sessions not at favourite charging station** | 14.87 | **0.009** | 15.44 | **0.007** | 15.09 | **0.008** |
| *Geographical factors* |  |  |  |  |  |  |
| **Paid parking** | 15.38 | **<0.001** | 15.55 | **<0.001** | 15.41 | **<0.001** |
| **Business per squared km** | -0.35 | **0.028** | -0.24 | 0.133 | -0.18 | 0.227 |
| **Houses per squared km** | 0.04 | 0.491 | 0.01 | 0.813 | 0.00 | 0.980 |
| **Income (€)**  *Facilities* | 0.35 | **<0.001** | 0.33 | **<0.001** | 0.32 | **<0.001** |
| **No of restaurants** | 0.37 | **0.001** | 0.43 | **0.009** | 15.41 | **<0.001** |
| **No of Pubs** | 0.65 | 0.098 | 0.75 | 0.152 | -0.18 | 0.227 |
| **No of Public transport stops** | 0.64 | **0.022** | 1.18 | **0.002** | 0.00 | 0.980 |
| **No of Shops** | -0.03 | 0.358 | -0.01 | 0.839 | 0.32 | **<0.001** |
| **Observations** | 1708 | | |  |  |  |
| **R^2^** | 0.186 |  | 0.184 |  | 0.183 |  |

**Appendix C: Multicollinearity and heteroskedasticity test results**

*Breusch-Pagan*

Average score: (χ2 = 0.2897073, Df = 1, p = 0.59041)

Energy: (χ2 = 1.362542, Df = 1, p = 0.2431)

Connection Time: (χ2 = 8.377158, Df = 1, p = 0.0037997)

Users: (χ2 = 124.9865, Df = 1, p = < 2.22e-16)

Sessions: (χ2 = 0.3980608, Df = 1, p = 0.52809 )

*Correlation table (same across all models):*

--- Figure 5 ----

**Figure 5 Correlation Table**

**Appendix D: Model specifications**

**Average Score = β _Year[2015]_ + β _Year [2016]_ + β _Year [2017]_ + β _Year [2018]_ + β _Year [2019]_ + β _Number of stations_+ β _Number of sessions_+ β _Number of users_+ β _Connection Time_+ β _Energy_ + β _Share of sessions not at favourite charging station_+ β _Paid parking_+ β _Business per squared km_+ β _Houses per squared km_+ β _Income (€)_+ β _No of restaurants_ + β _No of Pubs_ + β _No of Public transport stops_ + β _No of Shops_ + ε**

**Energy = β _Year[2015]_ + β _Year [2016]_ + β _Year [2017]_ + β _Year [2018]_ + β _Year [2019]_ + β _Number of stations_+ β _Number of sessions_+ β _Number of users_+ β _Connection Time_+ β _Energy_ + β _Share of sessions not at favourite charging station_+ β _Paid parking_+ β _Business per squared km_+ β _Houses per squared km_+ β _Income (€)_+ β _No of restaurants_ + β _No of Pubs_ + β _No of Public transport stops_ + β _No of Shops_ + ε**

**Connection Time= β _Year[2015]_ + β _Year [2016]_ + β _Year [2017]_ + β _Year [2018]_ + β _Year [2019]_ + β _Number of stations_+ β _Number of sessions_+ β _Number of users_+ β _Connection Time_+ β _Energy_ + β _Share of sessions not at favourite charging station_+ β _Paid parking_+ β _Business per squared km_+ β _Houses per squared km_+ β _Income (€)_+ β _No of restaurants_ + β _No of Pubs_ + β _No of Public transport stops_ + β _No of Shops_ + ε**

**Users = β _Year[2015]_ + β _Year [2016]_ + β _Year [2017]_ + β _Year [2018]_ + β _Year [2019]_ + β _Number of stations_+ β _Number of sessions_+ β _Number of users_+ β _Connection Time_+ β _Energy_ + β _Share of sessions not at favourite charging station_+ β _Paid parking_+ β _Business per squared km_+ β _Houses per squared km_+ β _Income (€)_+ β _No of restaurants_ + β _No of Pubs_ + β _No of Public transport stops_ + β _No of Shops_ + ε**

**Sessions = β _Year[2015]_ + β _Year [2016]_ + β _Year [2017]_ + β _Year [2018]_ + β _Year [2019]_ + β _Number of stations_+ β _Number of sessions_+ β _Number of users_+ β _Connection Time_+ β _Energy_ + β _Share of sessions not at favourite charging station_+ β _Paid parking_+ β _Business per squared km_+ β _Houses per squared km_+ β _Income (€)_+ β _No of restaurants_ + β _No of Pubs_ + β _No of Public transport stops_ + β _No of Shops_ + ε**
